# An interpretable machine learning approach for predicting drug-resistant epilepsy in children with tuberous sclerosis complex

**DOI:** 10.3389/fneur.2025.1623212

**Published:** 2025-08-04

**Authors:** Jie Fu, Genfu Zhang, Zhixian Yang, Jiong Qin

**Affiliations:** ^1^Department of Pediatrics, Peking University People’s Hospital, Beijing, China; ^2^Epilepsy Center, Peking University People’s Hospital, Beijing, China

**Keywords:** machine learning, model interpretability, predictive model, tuberous sclerosis complex, drug-resistant epilepsy

## Abstract

**Background:**

This study developed and validated an interpretable machine learning (ML) algorithm for predicting the risk of drug-resistant epilepsy (DRE) in children with Tuberous sclerosis (TSC).

**Methods:**

To estimate the risk of DRE in pediatric TSC patients, an interpretable ML model was developed and validated. Clinical data were retrospectively collected from 88 pediatric patients with TSC-related epilepsy. 9 ML algorithms were applied, such as random forest (RF), to construct predictive models. To improve interpretability, SHapley Additive exPlanations (SHAP) were employed, providing both global and individualized feature importance explanations.

**Results:**

The RF model outperformed all other algorithms, yielding an AUC of 0.862 and a specificity of 0.930. Key predictors of DRE included a history of infantile epileptic spasms syndrome (IESS), multifocal discharges on EEG, three or more cortical tubers, and the use of three or more antiseizure medications (ASMs). The model was further evaluated using tenfold cross-validation and showed good calibration and clinical utility, as confirmed by decision curve analysis (DCA).

**Conclusion:**

The RF-based prediction model provides a valuable tool for early identification of children with TSC at high risk for DRE, supporting individualized treatment decisions. The integration of SHAP improves model transparency and enhances clinical interpretability.

## 1 Introduction

Tuberous sclerosis complex (TSC) is a rare genetic disorder involving multiple organ systems, with a prevalence of around 1 in 6,000 to 1 in 10,000 individuals (1). More than 85% of TSC cases are associated with pathogenic variants in the TSC1 or TSC2 genes; however, 10–15% of clinically diagnosed cases show no detectable mutations (2). Genetic alterations in the TSC1 or TSC2 genes can result in the inactivation of their encoded proteins, leading to hyperactivation of the mTOR signaling pathway. This dysregulation compromises cellular and neuronal development, resulting in benign tumors across multiple organs and diverse neuropsychiatric manifestations (3–5).

Epilepsy is one of the most common neurological symptoms in individuals with TSC. About 70–90% of patients with TSC experience seizures (6, 7). The therapeutic goal in these patients is to achieve seizure freedom, thereby improving neurological and cognitive outcomes. In recent years, there has been a gradual shift from traditional treatment approaches toward more proactive strategies. Traditional approaches primarily involve the administration of antiseizure medications (ASMs) after seizures onset, while new strategies focus on preemptive treatment, such as using vigabatrin (VGB) or molecular targeted therapy with mTOR inhibitors (8, 9). However, approximately 60% of TSC-related seizures are drug-resistant, and the diagnosis of drug-resistant epilepsy (DRE) is often delayed (10). DRE imposes a significant burden on patients’ cognitive development, family life, and social functioning. Therefore, developing a predictive model is essential for the early recognition of individuals at elevated risk for developing DRE.

Previous studies have identified several predictive factors for drug resistance, including early-onset seizures, a prior diagnosis of infantile epileptic spasm syndrome (IESS), pathogenic mutations in the TSC2 gene, interictal epileptiform discharges on the electroencephalogram (EEG), and the presence of multiple cortical tubers (11–15). Due to the limited number of pediatric TSC patients, research on predicting epilepsy treatment outcomes in this population remains scarce, and most studies have only applied certain machine learning (ML) techniques. For example, Zhao et al. constructed a multilayer perceptron model that integrated 35 multimodal features including EEG, magnetic resonance imaging (MRI), genetic, and clinical data and achieved an AUC of 0.812 in predicting treatment outcomes (16). Shrot et al. used a random forest (RF) model based on structural imaging and clinical features to predict seizures and neurocognitive outcomes. However, its performance in seizure prediction was suboptimal, with the area under the receiver operating characteristic curve (AUC) values of 0.54 ± 0.19 in the training dataset and 0.71 in internal validation (17). In addition, Wang et al. developed a multi-technique deep learning method called WAE-Net for 300 children with TSC-related epilepsy, combining clinical data with multi-contrast MRI, including the combination of T2WI and FLAIR images into FLAIR3. This model reported a peak AUC of 0.908 in the test cohort (18). These studies highlight the potential of machine learning approaches in predicting treatment outcomes in TSC-related epilepsy. However, many of these models rely on multisequence MRI and complex deep learning architectures and often lack model interpretability. Therefore, there is an urgent clinical need for a predictive model that is structurally simple, highly interpretable, and based on routinely available clinical features to enable early identification and personalized intervention for DRE in children with TSC.

This study developed and validated an interpretable ML algorithm for predicting the risk of DRE in children with TSC. To enhance transparency and clinical applicability, the final model is interpreted using the SHapley Additive exPlanation (SHAP) method. This gives children with TSC a scientific foundation for early intervention and personalized treatment.

## 2 Method

### 2.1 Participants in the study

This study retrospectively analyzed clinical data from TSC patients admitted to the pediatric department at Peking University People’s Hospital between January 2018 and March 2024. The following were the criteria for inclusion: ① Diagnosis complied with the 2021 criteria proposed by the International Consensus Group for TSC (8); ② Epilepsy was diagnosed based on the guidelines issued by the International League Against Epilepsy (ILAE); ③ Availability of complete medical history, EEG, and cranial MRI or computerized tomography (CT) imaging; ④ A minimum follow-up duration of 1 year. Patients who did not fulfill these criteria were excluded from the study. During the follow-up period, the efficacy of ASM treatment was assessed in each patient with TSC-related epilepsy. This study adopted the 2010 definition of DRE proposed by ILAE (19). Patients were categorized as having DRE if they failed to achieve sustained seizure freedom after adequate trials of two or more tolerated and appropriately chosen ASMs. Sustained seizure freedom was defined as a seizure-free period of at least 12 months or three times the longest pre-treatment interseizure interval, whichever was longer. Patients who remained completely seizure-free for this duration were categorized into the seizure-free group. This retrospective cohort study was approved by the hospital’s ethics committee. All patient information was anonymized, and the need for informed consent was waived.

### 2.2 Clinical data and features collection

Medical and demographic data, including sex, age of onset, family history, and identified genetic variants, were retrospectively obtained from electronic medical records. Clinical features such as seizure types (focal seizures [FS] only, epileptic spasms [ES] only, FS combined with ES, generalized seizures), presence of (IESS, EEG findings, MRI/CT imaging, and number of ASMs) used were also collected. EEG data were recorded using the international standard 10–20 system (Neurofax; Nihon-Kohden, Tokyo, Japan) through 4-h video-electroencephalogram (VEEG) monitoring, encompassing at least one complete wake–sleep–wake cycle. All EEGs were independently evaluated by two experienced neurophysiologists. Developmental delay or cognitive impairment was determined based on neuropsychological assessments conducted by experienced neuropsychologists. These assessments covered attention, memory, motor skills, executive functions, visual perception, language abilities, and emotional regulation. Formal diagnoses of other neuropsychiatric disorders, such as autism spectrum disorder (ASD) or psychiatric comorbidities (e.g., anxiety, depression, and psychosis), were limited and therefore not included in the present analysis.

### 2.3 Variable selection and model development

We applied the recursive feature elimination (RFE) method to select variables. RFE is a widely used machine learning technique based on feature subset selection (20, 21). It iteratively eliminates features with lower contribution during the training process, ultimately identifying the most informative subset of features to achieve optimal model performance. During the feature selection process, 10-round 10-fold cross-validation was used to evaluate model performance. This repeated cross-validation approach facilitates a comprehensive assessment of model robustness and improves the reliability of the feature selection results.

This study used 9 ML models, including RF, support vector machine (SVM), gradient boosting machine (GBM), extreme gradient boosting (XGB), naive bayes (NB), k-nearest neighbor (KNN), neural network (NNET), decision tree (DT), and logistic regression (LR). RF is an ensemble bagging method known for its high accuracy and ability to handle missing data. SVM constructs optimal classification boundaries and performs well with small datasets. GBM and its optimized variant XGB iteratively reduce residual errors, with XGB offering enhanced scalability and regularization. NB based on probabilistic reasoning, is efficient for small, noisy datasets. KNN is a nonparametric algorithm that classifies based on local data similarity. NNET capture nonlinear relationships and integrate complex feature patterns. DT offers interpretable rule-based outputs but may overfit without pruning. LR remains a widely accepted, fast, and interpretable linear model, particularly suitable for clinical prediction when multicollinearity is addressed. Together, these models represent a spectrum of predictive paradigms suited for clinical applications. These classifiers were selected to represent a spectrum of modeling complexity, interpretability, and applicability in clinical prediction tasks. To optimize model performance, hyperparameter tuning was performed within the best feature subset for each model using repeated 10-fold cross-validation (10 repeats). The “caret” package was used with its default grid search settings (Supplementary Table 1). Final models were retrained on the training set using the selected features and optimal parameters.

### 2.4 Evaluation and comparison of model performance

Model performance was systematically assessed through standard evaluation metrics, such as, including the AUC, positive predictive value (PPV), negative predictive value (NPV), sensitivity, specificity, accuracy, Kappa coefficient, and Youden’s index. Calibration was assessed via the Hosmer–Lemeshow test, with calibration curves generated to visualize the agreement between predicted and observed outcomes. Decision curve analysis (DCA) is a statistical method used to evaluate the clinical utility of predictive models in real-world decision-making. It assesses the net benefit of different strategies across a range of threshold probabilities. Net benefit is defined as the difference between the proportion of true positives and the proportion of false positives or false negatives, weighted by the clinical consequences of each (22). Based on these assessments, the optimal ML model for predicting the risk of DRE in pediatric TSC patients was identified.

### 2.5 Model explanation

The interpretability of ML models is a key factor in their clinical applicability. However, complex models often suffer from the “black box” problem, which limits their practical application in clinical settings. To enhance model transparency and interpretability, this study employed the SHAP method to interpret prediction outcomes. By calculating SHAP values, the method visually illustrates the magnitude and direction of each feature’s impact on individual predictions. Global explanations evaluate the relative importance of features across the entire dataset, whereas local explanations reveal the specific factors contributing to individual predictions, thereby improving model transparency and interpretability (23).

### 2.6 Statistical analysis

R version 4.4.1 was utilized for data processing and statistical analysis. Continuous variables exhibiting a normal distribution are expressed as mean ± standard deviation (SD), and group comparisons were conducted utilizing the independent samples *t*-test. For non-normally distributed variables, the median and interquartile range (IQR) were used, and the Mann–Whitney U test was applied for comparisons. The chi-square test was used to assess group differences for categorical variables, which are represented as frequencies and percentages (%). When the chi-square test assumptions were violated, Fisher’s exact test was utilized. A *p*-value < 0.05 (two-tailed) was considered statistically significant.

To construct a conventional logistic regression model, univariate logistic regression was initially performed to identify potential predictors (*p* < 0.05). Significant variables were subsequently incorporated into a multivariate logistic regression model via a bidirectional stepwise selection approach. A nomogram was developed to visualize the final prediction model, which incorporated variables with statistically significant *p*-values (< 0.05) from the multivariate analysis. The Hosmer–Lemeshow test was used to assess the model’s calibration (*p* > 0.05 indicated a good fit), and calibration curves were produced using 1,000 bootstrap samples. Nomogram construction and calibration curve plotting were performed using the “rms” package. ROC analysis was conducted using the “pROC” and “ggplot2” packages, while bootstrap validation was implemented via the “caret” package. DCA was conducted using the “ggDCA” package, assessing net clinical benefit across threshold probabilities from 0.01 to 0.99.

Machine learning models were developed using the “caret” (version 6.0.94) package in R, which provides a unified interface for algorithm training, hyperparameter tuning, and performance evaluation. Nine algorithms were implemented, including RF (method set to “rf”), SVM (method set to “svmRadial”), GBM (method set to “gbm”), XGB (method set to “xgbTree”), NB (method set to “naive_bayes”), KNN (method set to “knn”), NNET (method set to “nnet”), DT (method set to “rpart”) and LR (method set to “glm”). Visualization of model performance was conducted with the “runway” package.

## 3 Results

### 3.1 Patient characteristics

**Figure 1:**
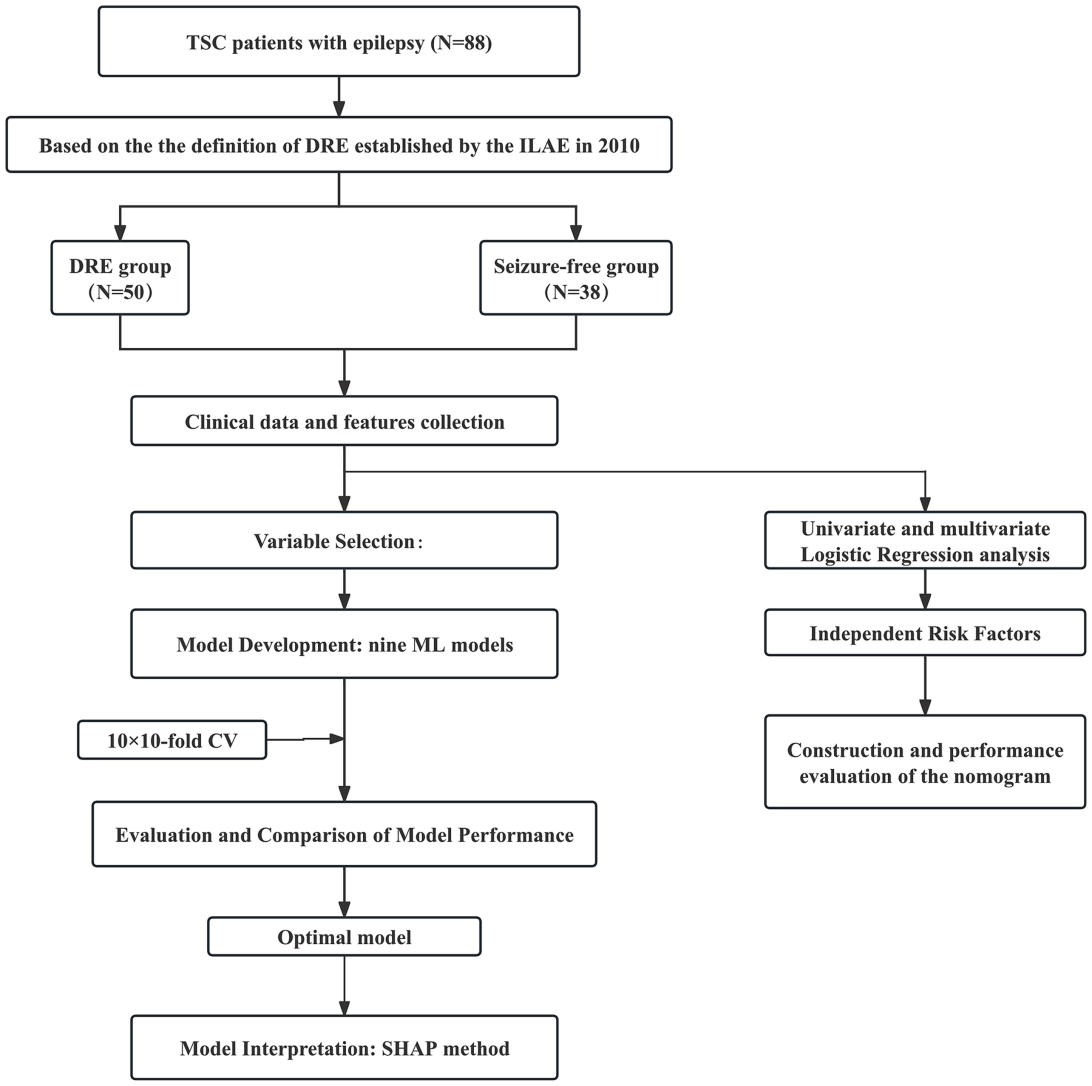
Flow diagram of the study design. TSC, tuberous sclerosis complex; DRE, drug-resistant epilepsy; CV, cross-validation; CT, computed tomography; MRI, magnetic resonance imaging; ML, machine learning; RF, random forest; KNN, k-nearest neighbors; DT, decision tree; SVM, support vector machine; NB, naive bayes; GBM, gradient boosting machine; NNET, neural network; XGB, extreme gradient boosting; LR, logistic regression; ROC, receiver operating characteristic; AUC, area under the receiver operating characteristic curve; DCA, decision curve analysis.

Among the 88 patients, 50 (56.8%) were classified as having DRE, while 38 (43.2%) achieved seizure freedom with medication. The median age at seizure onset was 13 months in the seizure-controlled group and 8 months in the DRE group. Genetic testing was conducted in 73 patients, of which 19 (21.6%) had TSC1 mutations, and 54 (61.4%) had TSC2 mutations. No statistically significant difference was seen in the distribution of genetic mutations between the two groups. 35 cases (39.8%) had a prior diagnosis of IESS, and 28 of them subsequently developed DRE. Approximately two-thirds of TSC patients exhibited varying degrees of psychomotor developmental delay. Furthermore, significant differences in clinical characteristics, EEG, and neuroimaging findings were observed between the two groups. For example, compared with the seizure-free group, patients with DRE had an earlier age of epilepsy onset (8 months vs. 13 months), a higher prevalence of IESS history (56% vs. 18.4%), and were more likely to exhibit ES (38% vs. 28.9%) or focal seizures combined with ES (20% vs. 2.6%). EEG findings in the DRE group revealed a higher frequency of interictal multifocal discharges (46% vs. 5.3%). Neuroimaging findings showed a greater number of cortical tubers (≥3: 88% vs. 63.2%) and a higher prevalence of subependymal nodules (SEN) (76% vs. 55.3%). The use of mTOR inhibitors was significantly lower in the DRE group compared to the seizure-free group (56% vs. 76.3%). All differences were statistically significant (*p* < 0.05). Table 1 describes the demographic and clinical characteristics of all patients. The study design is illustrated in Figure 1.

**Table 1:** Comparison of clinical and demographic characteristics between drug-resistant epilepsy (DRE) and seizure-free patients.

Characteristic	DRE (*n* = 50)	Seizure-free (*n* = 38)	*p* value
Sex, *n* (%)			0.739
Female	18 (36.0)	15 (39.5)	
Male	32 (64.0)	23 (60.5)	
Age of unset, Median (Q1, Q3)	8.00 (6.00, 12.00)	13.00 (8.50, 24.00)	0.005
Genotype, *n* (%)			0.843
TSC1	10 (20.0)	9 (23.7)	
TSC2	32 (64.0)	22 (57.9)	
NMI/ND	8 (16.0)	7 (18.4)	
Family history, *n* (%)			0.608
No	44 (88.0)	32 (84.2)	
Yes	6 (12.0)	6 (15.8)	
IESS, *n* (%)			< 0.001
No	22 (44.0)	31 (81.6)	
Yes	28 (56.0)	7 (18.4)	
Seizure type at onset, *n* (%)			0.020
Focal	19 (38.0)	24 (63.2)	
ES	19 (38.0)	11 (28.9)	
Focal+ES	10 (20.0)	1 (2.6)	
Generalized	2 (4.0)	2 (5.3)	
EEG findings, *n* (%)			< 0.001
Normal	1 (2.0)	8 (21.1)	
Focal	16 (32.0)	22 (57.9)	
Multifocal	23 (46.0)	2 (5.3)	
Generalized	10 (20.0)	6 (15.8)	
Multiple cortical tubers (≥3), *n* (%)			0.006
No	6 (12.0)	14 (36.8)	
Yes	44 (88.0)	24 (63.2)	
SENs, *n* (%)			0.040
No	12 (24.0)	17 (44.7)	
Yes	38 (76.0)	21 (55.3)	
SEGAs, *n* (%)			1.000
No	48 (96.0)	37 (97.4)	
Yes	2 (4.0)	1 (2.6)	
Number of ASMs, *n* (%)			< 0.001
<3	11 (22.0)	24 (63.2)	
≥3	39 (78.0)	14 (36.8)	
mTOR inhibitors, *n* (%)			0.048
No	22 (44.0)	9 (23.7)	
Yes	28 (56.0)	29 (76.3)	
Developmental delay, *n* (%)			0.119
No	18 (36.0)	20 (52.6)	
Yes	32 (64.0)	18 (47.4)	

NMI, no mutation identified; ND, not done; IESS, infantile epileptic spasms syndrome; ES, epileptic spasms; EEG, electroencephalogram; SEN, subependymal nodule; ASM, anti-seizure medication; mTOR, mechanistic target of rapamycin; SEGA, subependymal giant cell astrocytoma.

**Figure 2:**
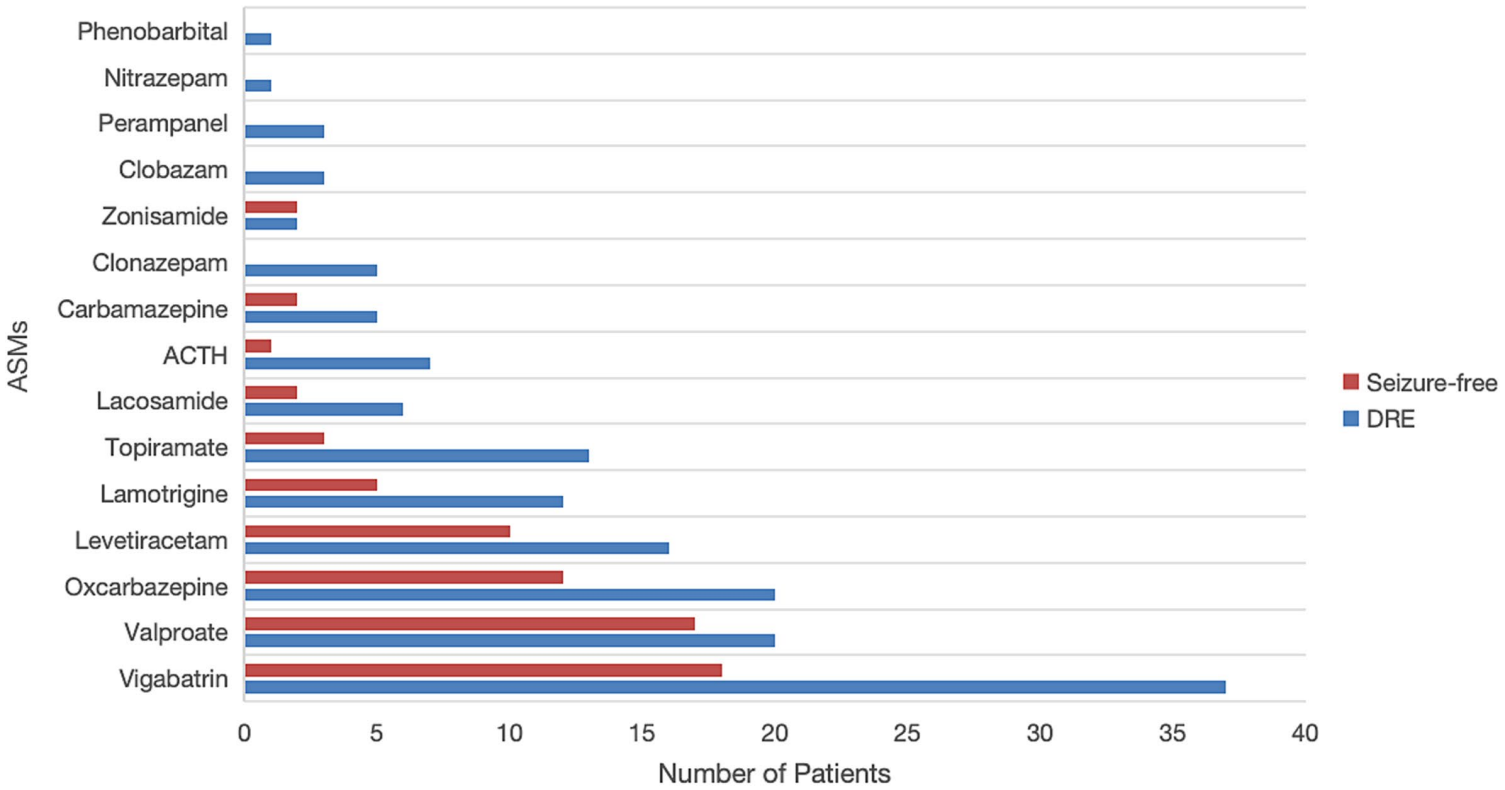
Comparison of commonly used antiseizure medications (ASMs) between drug-resistant epilepsy (DRE) and seizure-free patients. ACTH, adrenocorticotropic hormone.

Figure 2 illustrates the use of ASMs among 88 patients with TSC in our study. All patients received at least one ASM, with the five most commonly prescribed being vigabatrin (62.5%), valproate (42.0%), oxcarbazepine (36.4%), levetiracetam (29.5%), and lamotrigine (19.3%). Notably, patients in the DRE group tended to receive a broader variety and higher number of ASMs, as detailed in Supplementary Table 2.

In addition to ASMs, 3 patients (3.4%) received a ketogenic diet, 5 patients (5.7%) underwent epilepsy surgery (with 3 achieving seizure freedom), and 1 patient (1.1%) received vagal nerve stimulation (VNS) as non-pharmacologic interventions.

### 3.2 Independent risk factor analysis

Based on the full cohort, potential risk factors for DRE in patients with TSC were explored. Univariate logistic regression analysis (*p* < 0.05) identified 8 factors potentially associated with DRE, including age at seizure onset, history of IESS, seizure type (focal seizures combined with ES), EEG findings of interictal multifocal discharges, cortical tubers ≥3, presence of SENs, and use of ≥3 number of ASMs (Supplementary Table 3). Multivariate logistic regression with stepwise selection was conducted to explore independent predictors of DRE in individuals with TSC. 4 variables were identified as independent risk factors for DRE, with statistical significance (*p* < 0.05). These included: history of IESS (OR = 22.987, 95% CI: 1.858–34.651, *p* = 0.007), EEG findings of interictal multifocal discharges (OR = 7.139, 95% CI: 1.927–671.336, *p* = 0.027), presence of multiple cortical tubers (OR = 6.265, 95% CI: 1.404–34.991, *p* = 0.023), and use of ≥3 number of ASMs (OR = 9.469, 95% CI: 2.569–44.156, *p* = 0.002) (Table 2).

**Table 2:** Stepwise multivariate logistic regression analysis of risk predictors for drug-resistant epilepsy (DRE) in pediatric tuberous sclerosis complex (TSC) patients.

Characteristic	*β*	SE	*z*	OR (95% CI)	*p* value
IESS	1.966	0.733	2.682	7.139 (1.858, 34.651)	0.007
EEG findings					
Focal	0.430	1.273	0.338	1.537 (0.156, 35.655)	0.735
Multifocal	3.135	1.413	2.218	22.987 (1.927, 671.336)	0.027
Generalized	0.805	1.396	0.576	2.236 (0.173, 60.681)	0.564
Multiple cortical tubers	1.835	0.806	2.275	6.265 (1.404, 34.991)	0.023
Number of ASMs≥3	2.248	0.712	3.156	9.469 (2.569, 44.156)	0.002

IESS, infantile epileptic spasms syndrome; EEG, electroencephalogram; ASM, anti-seizure medication.

### 3.3 Construction and performance evaluation of the nomogram

**Figure 3:**
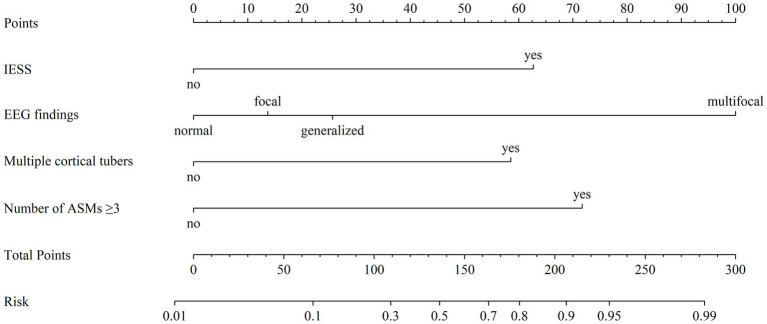
Nomogram model for predicting the risk of drug-resistant epilepsy (DRE) in pediatric tuberous sclerosis complex (TSC) patients. For each predictor, locate the corresponding value and draw a vertical line upward to determine its individual point value on the “Points” axis. Sum the points across all predictors to obtain a “Total Points” score. This total is then mapped downward to the “Risk” axis to estimate the probability of developing DRE. “Multiple cortical tubers” refers to cases with ≥3 cortical tubers. EEG, electroencephalogram; ASMs, anti-seizure medications; IESS, infantile epileptic spasm syndrome.

**Figure 4:**
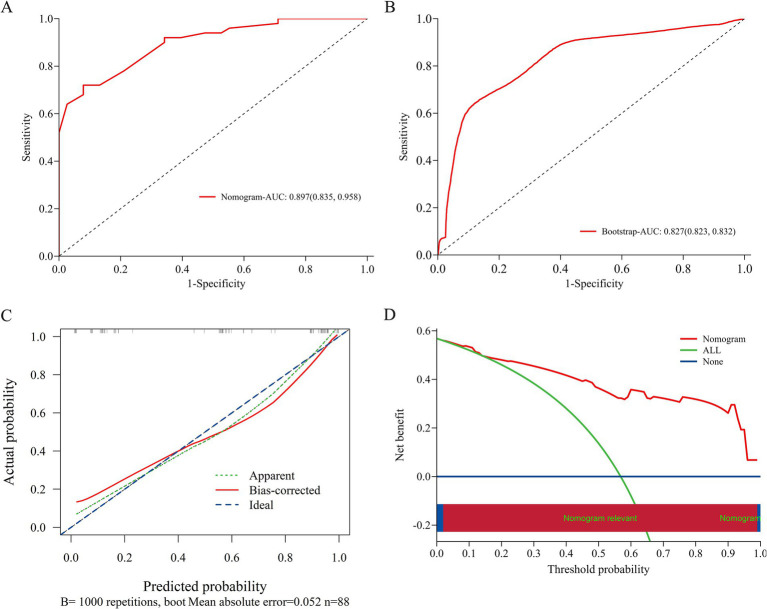
Evaluation and validation of the nomogram model. **(A)** ROC curve of the original nomogram model. The AUC of the model was 0.897 (95% CI, 0.835–0.958). **(B)** ROC curve after internal validation using 1,000 bootstrap resamples. The AUC with 1,000 Bootstrap resampling was 0.827 (95% CI, 0.823–0.832). **(C)** Calibration curve of the nomogram model; The x-axis represents the predicted probability of DRE, and the y-axis represents the observed probability. The ideal diagonal line represents perfect concordance between predicted and observed outcomes. The apparent line shows the model’s original performance, while the bias-corrected line reflects its performance after correction for potential overfitting via 1,000 bootstrap resamples. The calibration curve demonstrates good agreement between predicted and observed probabilities, indicating that the nomogram is well-calibrated under internal validation. **(D)** DCA curve of the nomogram model; The x-axis displays the threshold probability, and the y-axis represents the net clinical benefit. DCA, decision curve analysis; AUC, area under the receiver operating characteristic curve; ROC, receiver operating characteristic.

A nomogram was developed based on the results of conventional logistic regression analysis, incorporating the following predictors: history of IESS, EEG findings, presence of multiple cortical tubers, and the number of ASMs used. DRE was defined as the outcome variable (Figure 3). The AUC of the model was 0.897 (95% CI, 0.835–0.958, *p* < 0.001) (Figure 4A). Internal validation was carried out through 1,000 bootstrap resamples. Following internal validation, the AUC of the nomogram was 0.827 (95% CI, 0.823–0.832) (Figure 4B), demonstrating good predictive performance and stability. Following internal validation, the calibration curve of the nomogram was generated. With a mean absolute error (MAE) of 0.052, the model demonstrated good agreement between predicted and observed outcomes (Figure 4C). As shown in the DCA curve (Figure 4D), when the threshold probability exceeded 0.2, the nomogram demonstrated a higher net clinical benefit than either the “treat-all” or “treat-none” strategies, supporting its potential clinical utility in predicting DRE.

### 3.4 Variable selection for prediction

This study employed the RFE method for variable selection, aiming to identify the subset of variables that contributes most significantly to model performance. Supplementary Figure 1 shows the RFE-based feature selection process for each ML model. Supplementary Figure 2 shows a bar chart representing the calculated significance scores of the chosen features, reflecting their relative contributions to model prediction.

### 3.5 Construction and performance comparison of models

**Figure 5:**
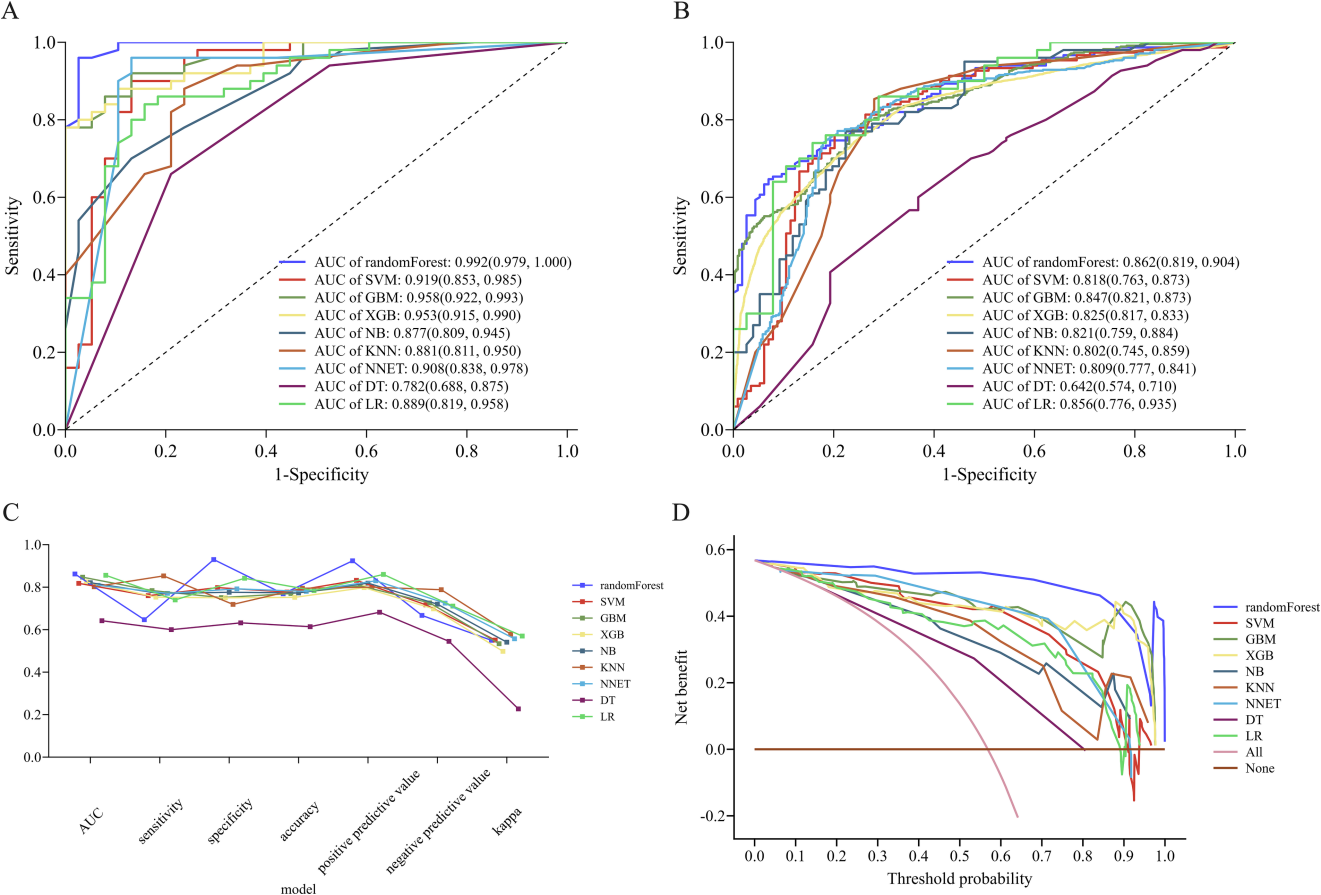
Performance of nine machine learning (ML) models. **(A)** ROC curve analysis of the 9 ML models. **(B)** ROC curve from internal cross-validation. **(C)** Parallel line graph comparing evaluation metrics across models, and **(D)** DCA curves for each model. ROC, receiver operating characteristic; AUC, area under the receiver operating characteristic curve; DCA, decision curve analysis; RF, random forest; SVM, support vector machine; KNN, K-nearest neighbors; NB, naive bayes; XGB, extreme gradient boosting; GBM, gradient boosting machine; NNET, neural network; DT, decision tree; LR, logistic regression.

We constructed 9 ML models using 10-fold cross-validation repeated 10-fold CV. Figures 5A and B depict the ROC curves of the models prior to and following internal cross-validation, respectively. Supplementary Table 4 provides detailed performance metrics of all models. The RF model achieved the highest specificity and AUC, recording an AUC of 0.862 (95% CI: 0.819–0.904) and a specificity of 0.930 (95% CI: 0.883–0.977). The GBM model followed, recording an AUC of 0.847 (95% CI: 0.821–0.873) and a specificity of 0.751 (95% CI, 0.706–0.797). The SVM model ranked third, achieving an AUC of 0.818 (95% CI, 0.763–0.873) and a specificity of 0.798 (95% CI, 0.725–0.872) (Figures 5B,C).

In terms of model consistency evaluation, the RF model exhibited the greatest Kappa value (0.550, 95% CI: 0.457–0.644), indicating substantial agreement between its predictions and actual outcomes. Kappa values for the SVM and GBM models were 0.551 (95% CI: 0.450–0.651) and 0.534 (95% CI: 0.474–0.593), respectively, suggesting comparable overall performance (Figure 5C). According to the Hosmer-Lemeshow test, the RF (*p* = 0.077) and SVM (*p* = 0.064) models exhibited a superior goodness-of-fit (Supplementary Figure 3). DCA revealed that the RF model achieved the greatest net clinical benefit throughout all threshold probabilities (0–1.0), followed by the XGB and GBM models (Figure 5D).

In summary, considering the combined evaluation metrics—including AUC, specificity, sensitivity, and model calibration (Hosmer–Lemeshow test)—the RF model demonstrated the best overall performance.

### 3.6 Model interpretation

**Figure 6:**
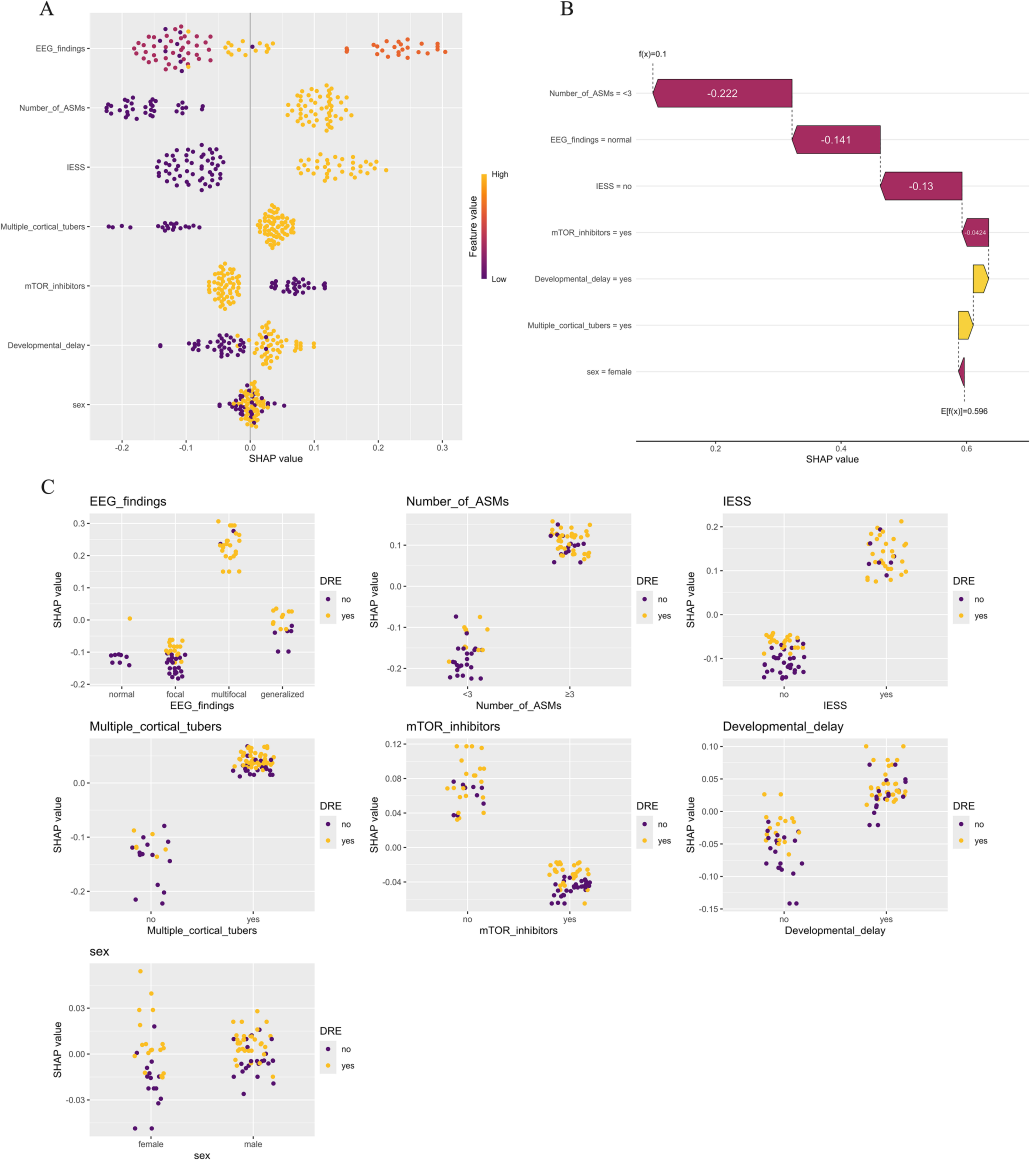
Model explanations using the SHapley Additive exPlanation (SHAP) method. **(A)** SHAP summary plot illustrating the influence of various features on the risk of drug-resistant epilepsy (DRE). Each point denotes the SHAP value of a specific feature for an individual, with orange representing higher feature values and purple lower ones. Vertical clustering indicates the distribution density of data points. **(B)** SHAP waterfall plot depicting how individual features contributed to the RF model’s prediction for a seizure-free patient with tuberous sclerosis complex (TSC). Orange bars indicate positive influence, while purple bars show negative impact. Notable features include the number of antiseizure medications (−0.222), EEG results (−0.141), prior infantile epileptic spasm syndrome (−0.130), and use of mTOR inhibitors (−0.0424). **(C)** SHAP dependence plot illustrating how an individual feature influences the model’s prediction, with each point corresponding to one patient. The x-axis shows actual feature values, while the y-axis represents SHAP values. Features with SHAP values > 0 increase the predicted likelihood of DRE.

Based on validation results, the RF model, which demonstrated the highest overall predictive performance, was selected for SHAP-based interpretability analysis. This interpretability methods offers both global feature-level and local patient-level explanations, thereby enhancing clinical interpretability. The SHAP summary plot (Figure 6A) visually illustrates both the direction and magnitude of each feature’s impact on model predictions. EEG findings have the greatest impact on the prediction model for DRE in TSC children. Specifically, the presence of multifocal or generalized discharges during the interictal period was correlated with an increased predicted risk of DRE. Other important risk factors included a heightened number of ASMs, a history of IESS, and an increased number of cortical tubers, all of which contributed positively to the model’s DRE risk prediction. Figure 6B demonstrates the contribution of each variable to the predicted outcome of a TSC patient with seizure freedom, as generated by the RF model. Figure 6C illustrates the relationship between the actual values of 7 features and their corresponding SHAP values. Features with SHAP values above zero contribute positively to the predicted probability of DRE, indicating a stronger association with increased DRE risk. For example, interictal multifocal or generalized EEG discharges, the use of three or more ASMs, a history of IESS, and the presence of multiple cortical tubers (≥3) in TSC patients are all associated with SHAP values above zero, thereby shifting the model’s prediction toward the DRE category.

## 4 Discussion

Studies have demonstrated that early preventive use of ASMs has a beneficial effect in patients with TSC and may improve long-term cognitive outcomes (24). However, due to the lack of precise assessment of drug treatment outcomes, most TSC patients experience long-term failure of ASM treatment after diagnosis, leading to poor seizure control and eventually developing DRE (25). Previous research has reported that up to 62.5% of children with TSC and epilepsy develop DRE (6). In this study, 56.8% of epilepsy patients exhibited drug resistance, which is consistent with prior reports. Accurately predicting the therapeutic response to ASM treatment is critical not only for designing individualized treatment strategies but also for improving seizure outcomes and preserving neurological development. However, clinical symptoms and treatment response alone often fail to provide sufficient information for predicting treatment efficacy. Therefore, identifying children with TSC at high risk for DRE as early as possible remains a key clinical priority.

A total of 88 children diagnosed with TSC-associated epilepsy were enrolled in this 6-year cohort study. A predictive model for the risk of DRE in patients with TSC was developed by evaluating 9 ML algorithms, incorporating clinical, EEG, and neuroimaging features. Additionally, the SHAP method was applied to identify and interpret the most important predictive features and their individual contributions to the model’s output. Among the nine ML models evaluated in this study, the RF model achieved the highest AUC and demonstrated superior performance across key parameters including specificity, calibration, and net clinical benefit. Prior research has also highlighted the utility of the RF algorithm in medical predictive modeling (20, 26). By aggregating multiple decision trees and employing a voting mechanism, the RF algorithm enhances predictive accuracy and robustness. It is particularly well-suited for analyzing complex, nonlinear relationships within medical datasets. Recent studies have demonstrated the utility of RF models in neurological disorders. For example, RF-based classifiers achieved high accuracy in distinguishing temporal lobe epilepsy with hippocampal sclerosis using MRI volumetric data, and in detecting and monitoring Alzheimer’s disease and mild cognitive impairment through EEG biomarkers (27, 28). Additionally, the ensemble strategy of the RF model helps to mitigate the risk of overfitting commonly seen with individual decision trees (29).

Accurate feature selection is one of the most critical components in the development of clinical prediction models. Therefore, RFE method was employed to identify an optimal subset of features, resulting in a simplified and clinically applicable ML prediction model. In this study, a final RF model was constructed using 7 features that can be easily evaluated during routine follow-up of patients with TSC. This provides a practical tool for early identification and risk stratification of DRE in the TSC population.

Traditional multifactorial logistic regression analysis revealed that a history of IESS, multifocal discharges on EEG, the presence of ≥3 cortical tubers, and the use of ≥3 number of ASMs are independent predictors contributing to the onset of DRE in children with TSC and epilepsy. Based on these four variables, we developed a nomogram that achieved an AUC of 0.827, demonstrating good discriminative ability using only routinely available clinical data. While the nomogram’s AUC was lower than that of the RF model, its interpretability and user-friendly design make it a valuable complementary clinical tool in settings with limited access to real-time ML platforms or low-resource environments. The visual format of the nomogram facilitates bedside application and enhances the accessibility of individualized risk estimation, thereby expanding the predictive model’s clinical utility across diverse healthcare settings.

In this study, a history of IESS was shown to markedly raise the likelihood of DRE (OR = 7.139, 95% CI: 1.858–34.651). A cohort study involving 1,546 TSC patients with epilepsy reported similar findings, in which a history of IESS was strongly associated with an increased risk of DRE. Moreover, among 389 individuals with available IESS treatment outcomes, IESS that could not be controlled by medication, surgery, or dietary intervention significantly increased the risk of DRE (12). The significance of IESS in the development of DRE could be linked to the early occurrence of epilepsy, diagnostic issues, and the challenges associated with timely intervention and treatment (13).

This study found that patients with TSC who exhibited multifocal discharges on interictal EEG had a significantly increased risk of developing DRE. Similarly, De Ridder et al. found that children with multifocal interictal epileptiform discharges (IEDs) on their initial EEG were more likely to develop DRE than those with focal IEDs (30). They also hypothesized that individuals with multifocal IEDs on initial EEGs might benefit more from prophylactic anticonvulsant therapy. Recent research has recommended frequent EEG monitoring for all patients diagnosed with TSC. If asymptomatic epileptiform activity is detected on EEG prior to the onset of clinical seizures, immediate administration of VGB is recommended. The EPISTOP trial, conducted from 2014 to 2018 across 9 European centers and one in Australia, was designed to compare the safety and efficacy of standard epilepsy treatment with preventive VGB therapy. The study included neonates and infants under 4 months old who had not yet experienced a seizure. All participants underwent continuous EEG monitoring, and VGB treatment was initiated upon detection of interictal discharges or epileptic seizures, at a minimum daily dose of 100 mg/kg. Among the 25 children who received preventive VGB therapy, the onset of clinical seizures was significantly delayed compared with 25 children who started treatment after seizure onset, thereby reducing the risk of DRE and preventing the occurrence of IESS (9). In the EPISTOP trial, preventive therapy was linked to a significantly lower risk of DRE than conventional treatment, showing a more than two-fold difference (RCT: 28% vs. 64%) (30).

Furthermore, this study found that TSC patients with DRE had a greater number of cortical tubers and SENs. A cortical tuber load of ≥3 was found to be an independent variable associated with DRE. This finding aligns with previous studies, which suggest that a higher cortical tuber burden serves as a significant biomarker for more severe neurological phenotypes in patients with TSC (31–33). While earlier research has primarily examined the relationship between the number of tubers, the tuber-to-brain volume ratio, and neurological outcomes, recent research has emphasized the possible influence of “cyst-like” tubers and cerebellar tubers on disease severity (17).

This study utilized multiple methods to assess model performance, including the AUC-ROC curve, Hosmer–Lemeshow test, and calibration curves. Additionally, a clinically applicable nomogram was developed, and DCA demonstrated high practical value in identifying DRE associated with TSC. In the training set, the RF model achieved an AUC of 0.992, suggesting potential overfitting. In comparison, performing 10-fold cross-validation 10 times resulted in an average AUC value of 0.862, which indicates excellent predictive performance for clinical application.

Although complex ML models can yield highly accurate predictions, they often suffer from poor interpretability, creating the so-called “black box” problem. Another strength of this study is the integration of the SHAP method, which improves transparency by providing visual and quantitative explanations of how the model makes predictions. SHAP assigns each feature an importance value for a particular prediction, allowing clinicians to better understand the reasoning behind the output and to build trust in model-based decision support tools (23). In this study, global SHAP analysis identified multifocal EEG discharges, the number of cortical tubers, and history of IESS as the most influential predictors of drug resistance, aligning well with known clinical risk factors. Furthermore, local SHAP plots provided case-specific insights. For example, Figure 6B illustrates a patient who achieved seizure freedom. The SHAP waterfall plot shows that the low predicted risk was driven by favorable factors including fewer antiseizure medications, normal EEG, absence of IESS, and use of mTOR inhibitors. Such local explanations can aid in personalized decision-making by clarifying how individual features contribute to risk, even in complex clinical contexts.

In our cohort, the utilization rate of mTOR inhibitor therapy was higher in seizure-free patients (76%) than in those with DRE (56%). Although mTOR inhibitor use was not included in the final feature subset selected by the ML model, SHAP analysis revealed that this variable exerted a negative contribution to the predicted risk of DRE, suggesting its potential protective role. However, its predictive weight remained relatively low compared to core features such as multifocal EEG discharges or cortical tuber burden, which may be attributed to multiple factors including timing of intervention, clinical heterogeneity in treatment indications (e.g., use for subependymal giant cell astrocytoma), and the use of a binary variable (“ever received” treatment) that failed to capture treatment duration, dosage, or adherence. Mechanistically, mTOR inhibitors may improve brain structure by correcting neuronal morphological abnormalities, reducing cell volume, promoting myelination and synaptic plasticity, and lowering the levels of inflammatory mediators. Rapamycin reduced seizure frequency by ≥50% in 56% of pediatric DRE patients (aged 11 months–14 years) in an open-label study, with greater efficacy observed in those treated within 6 months of seizure onset (34). Currently, only everolimus, a rapamycin derivative, has prospective controlled data supporting its long-term use in TSC patients. For TSC-associated DRE, everolimus has been investigated as an adjunctive treatment. According to the EXIST-3 research, which assessed everolimus’s safety and effectiveness, it had a positive benefit–risk ratio and considerably decreased seizure frequency when used as an adjuvant medication (35, 36).

While ASMs remained the primary treatment modality, a small subset of patients also received non-pharmacologic interventions. These included ketogenic diet, epilepsy surgery, and VNS, which are recognized options for drug-resistant epilepsy. However, their limited use in our cohort may be attributed to factors such as patient age, suitability for surgery, or constraints in access to specialized care, which in turn could have reduced their influence on model performance and variable selection.

Compared to traditional logistic regression models, the ML approach offers several clinically meaningful advantages. First, instead of estimating average effects at the group level, it captures complex nonlinear relationships among clinical, EEG, and MRI features, enabling individualized prediction of DRE risk and supporting personalized treatment planning for children with TSC. Second, all input variables are routinely collected in standard clinical care, which improves the model’s feasibility and scalability without requiring advanced imaging techniques or molecular biomarkers. Third, SHAP-based interpretation enhances model transparency and provides additional insights by not only confirming well-established predictors such as multifocal discharges and cortical tuber burden but also identifying less prominent factors like mTOR inhibitor use. This predictive model can support clinical decisions at two key stages. First, at the time of TSC diagnosis, before seizures occur, it enables early identification of infants at high risk for drug-resistant epilepsy by using routine EEG and MRI data. These patients may benefit from early intervention with vigabatrin or mTOR inhibitors and more frequent EEG monitoring, consistent with current expert recommendations and the EPISTOP trial. Second, after the first clinical seizure, the model can help stratify risk promptly, guide timely adjustment of antiseizure medications, and support referral for surgical evaluation. In addition, the presence of high-risk features identified by the model may inform genetic counseling and assist families in anticipating the clinical course and planning care.

While this study employed classical ML algorithms to ensure interpretability and feasibility in clinical environments, emerging artificial intelligence approaches may further enhance predictive performance. For instance, Transformer-based deep learning models have recently been applied to predict individual responses to antiseizure medications using structured clinical data, demonstrating the potential for personalized treatment strategies in epilepsy management (37). Additionally, graph neural networks (GNNs), which incorporate spatial and topological information from electrode placement and brain imaging, have shown superiority over traditional neural networks in surgical planning for DRE (38). These advancements suggest that future research, particularly when supported by large and multimodal datasets, could explore these novel architectures to improve the accuracy and clinical applicability of DRE risk prediction in pediatric TSC patients.

This study has several limitations. First, its retrospective design and single center setting with a limited sample size (*n* = 88) may introduce selection bias and limit the generalizability of findings. Second, although repeated 10-fold cross-validation was applied to reduce overfitting, the discrepancy between internal training performance and cross-validation results suggests the model may have captured dataset-specific noise. Third, some potentially relevant variables, such as advanced neuroimaging features (e.g., tuber burden and anatomical distribution) and socioeconomic indicators were not included and may have impacted model accuracy. Fourth, comorbidities such as cardiac, renal, pulmonary complications and neuropsychiatric disorders were not analyzed due to incomplete and heterogeneous documentation in the retrospective records. Fifth, although EEGs were independently interpreted by two experienced neurophysiologists, inter-rater variability remains possible, particularly in broader clinical contexts. Future adoption of standardized EEG scoring systems or AI-based analysis may help improve reproducibility. Despite these limitations, the final predictive model demonstrated excellent performance and holds promise for clinical application.

## 5 Conclusion

In conclusion, this study successfully developed and validated an interpretable risk prediction model for DRE in children with TSC and epilepsy, using 9 machine learning algorithms and clinical data from 88 patients. Among the models tested, the RF model exhibited the highest predictive performance, with an AUC of 0.862. The model’s interpretability and transparency were further enhanced by the integration of SHAP analysis. This predictive tool may aid clinicians in the early identification of children with TSC who are at increased risk for DRE, thereby enabling earlier intervention and more individualized treatment strategies.

## Data Availability

The original contributions presented in the study are included in the article/Supplementary material, further inquiries can be directed to the corresponding authors.

## Supplementary material

SUPPLEMENTARY FIGURE 1 Depiction of recursive feature elimination (RFE) for nine machine learning models. Each plot displays cross-validation accuracy (y-axis) as a function of the number of variables (x-axis). **(A)** RF, Random Forest, **(B)** SVM, Support Vector Machine, **(C)** KNN, k-Nearest Neighbors, **(D)** NB, Naive Bayes, **(E)** XGB, Extreme Gradient Boosting, **(F)** GBM, Gradient Boosting Machine, **(G)** NNET, Neural Network, **(H)** DT, Decision Tree, **(I)** LR, Logistic Regression.

SUPPLEMENTARY FIGURE 2 Feature importance ranking from RFE. Feature importance scores for different models are shown, with higher scores indicating greater predictive contribution. **(A)** RF, Random Forest, **(B)** SVM, Support Vector Machine, **(C)** KNN, k-Nearest Neighbors, **(D)** NB, Naive Bayes, **(E)** XGB, Extreme Gradient Boosting, **(F)** GBM, Gradient Boosting Machine, **(G)** NNET, Neural Network, **(H)** DT, Decision Tree, **(I)** LR, Logistic Regression.

SUPPLEMENTARY FIGURE 3 Calibration curve of nine machine learning models. The figure displays the calibration curve of nine machine learning models. RF, Random Forest; SVM, Support Vector Machine; KNN, k-Nearest Neighbors; NB, Naive Bayes; XGB, Extreme Gradient Boosting; GBM, Gradient Boosting Machine; NNET, Neural Network; DT, Decision Tree; LR, Logistic Regression.
