# Supplementary figures and images for: An interpretable machine learning approach for predicting drug-resistant epilepsy in children with tuberous sclerosis complex
Source: Front Neurol. 2025 Aug 4;16:1623212. doi: 10.3389/fneur.2025.1623212 (PMC12358403; doi:10.3389/fneur.2025.1623212)

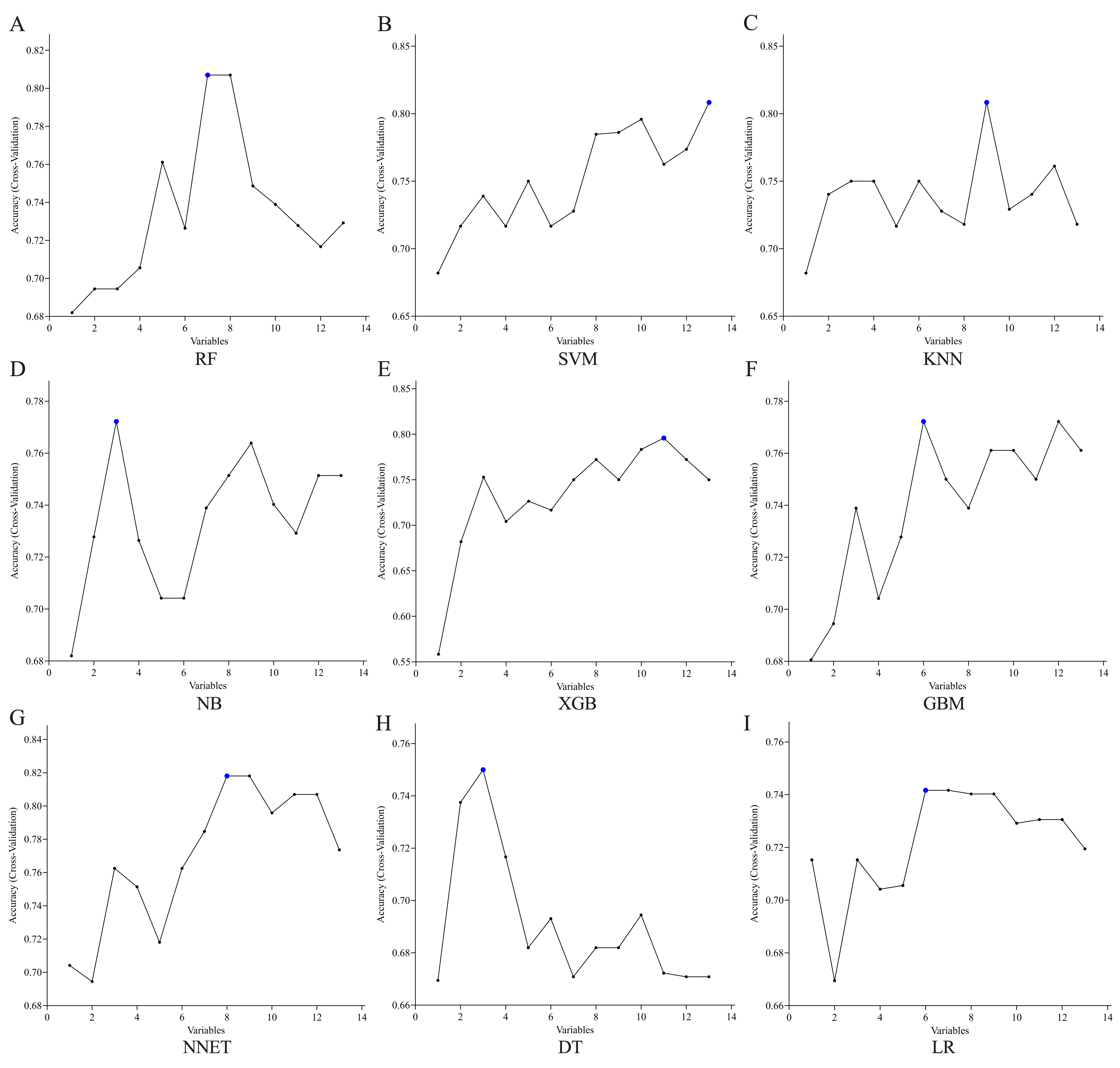

Supplement: SUPPLEMENTARY FIGURE 1 — Depiction of recursive feature elimination (RFE) for nine machine learning models. Each plot displays cross-validation accuracy (y-axis) as a function of the number of variables (x-axis). (A) RF, Random Forest, (B) SVM, Support Vector Machine, (C) KNN, k-Nearest Neighbors, (D) NB, Naive Bayes, (E) XGB, Extreme Gradient Boosting, (F) GBM, Gradient Boosting Machine, (G) NNET, Neural Network, (H) DT, Decision Tree, (I) LR, Logistic Regression. [file Image_1.tiff]

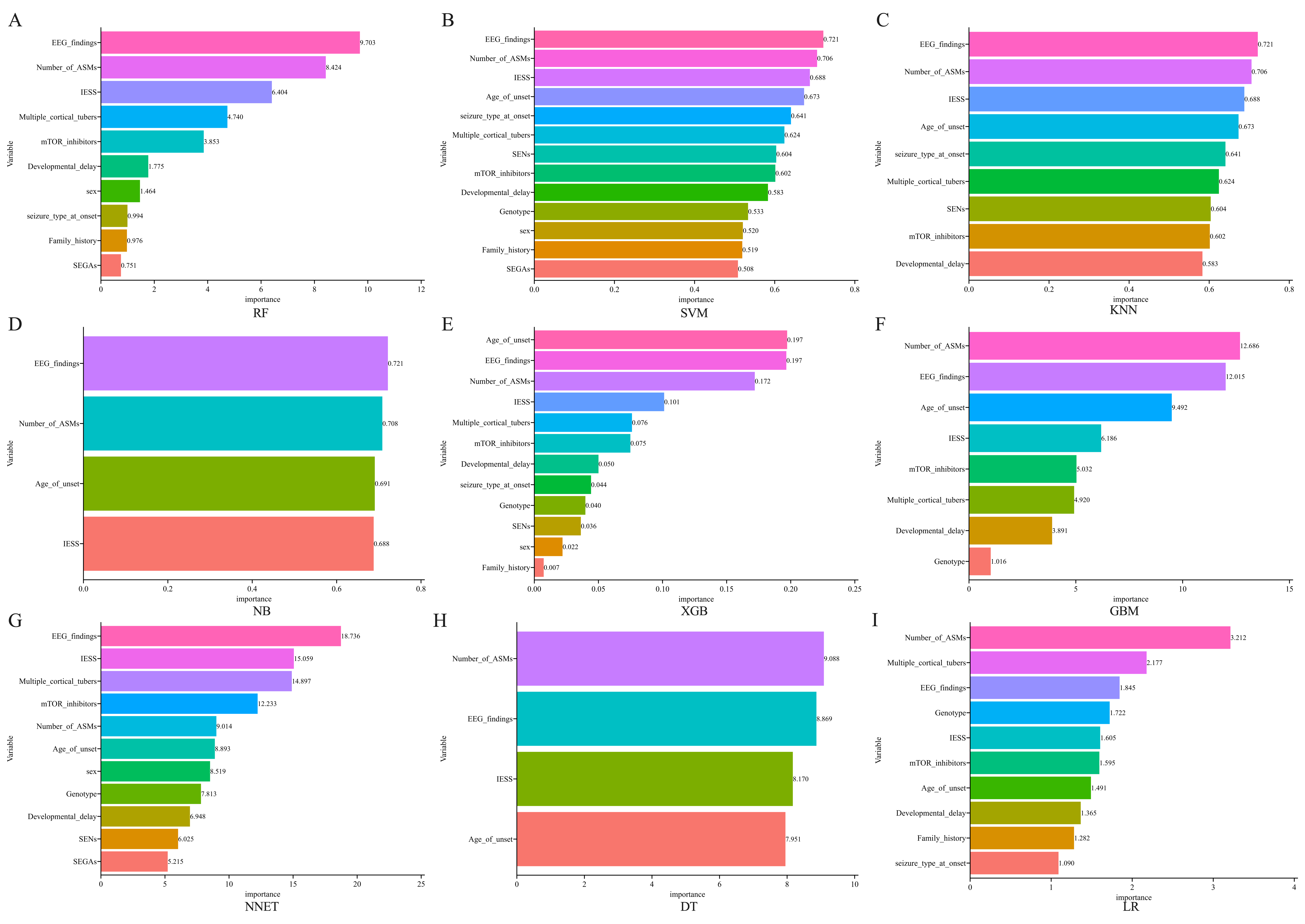

Supplement: SUPPLEMENTARY FIGURE 2 — Feature importance ranking from RFE. Feature importance scores for different models are shown, with higher scores indicating greater predictive contribution. (A) RF, Random Forest, (B) SVM, Support Vector Machine, (C) KNN, k-Nearest Neighbors, (D) NB, Naive Bayes, (E) XGB, Extreme Gradient Boosting, (F) GBM, Gradient Boosting Machine, (G) NNET, Neural Network, (H) DT, Decision Tree, (I) LR, Logistic Regression. [file Image_2.tiff]

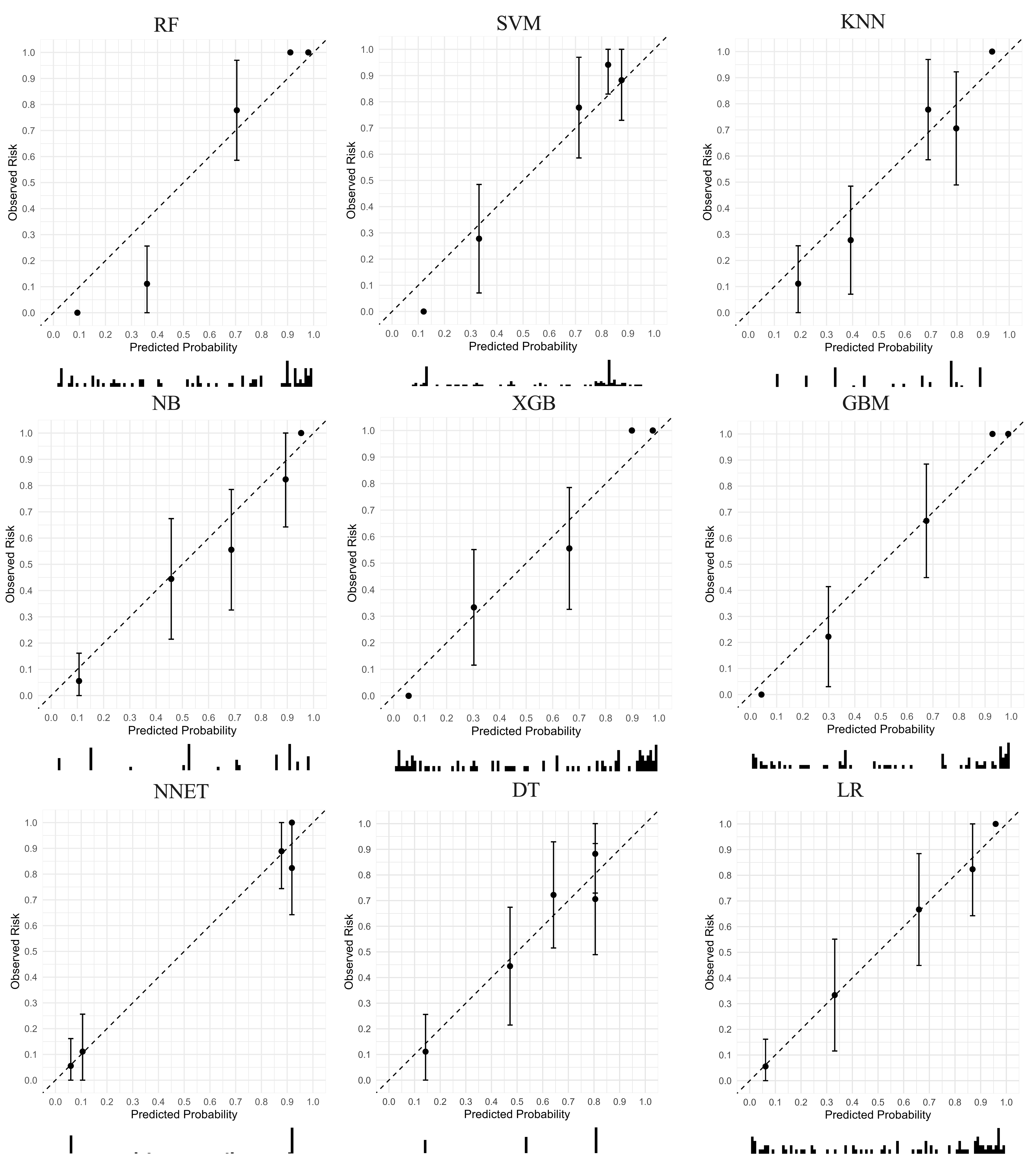

Supplement: SUPPLEMENTARY FIGURE 3 — Calibration curve of nine machine learning models. The figure displays the calibration curve of nine machine learning models. RF, Random Forest; SVM, Support Vector Machine; KNN, k-Nearest Neighbors; NB, Naive Bayes; XGB, Extreme Gradient Boosting; GBM, Gradient Boosting Machine; NNET, Neural Network; DT, Decision Tree; LR, Logistic Regression. [file Image_3.tiff]
