# Supplementary material for: An interpretable machine learning approach for predicting drug-resistant epilepsy in children with tuberous sclerosis complex
Source: Front Neurol. 2025 Aug 4;16:1623212. doi: 10.3389/fneur.2025.1623212 (PMC12358403; doi:10.3389/fneur.2025.1623212)
Supplement: Supplementary file 4 [file Table_1.docx]

Supplementary Material

**Supplementary Table 1. Hyperparameters for nine models**

| **Model** | **Hyperparameters** |
| --- | --- |
| Random Forest (RF) | mtry=2;min.node.siz=1,spitruel='gini' |
| Support Vector Machine (SVM) | sigma = 0.04318 and C = 1 |
| Gradient Boosting Machine (GBM) | n.trees = 150, interaction.depth = 2, shrinkage = 0.1 and n.minobsinnode = 10 |
| Extreme Gradient Boosting (XGB) | gama=0;nrounds = 100, max_depth = 1, eta = 0.3, gamma = 0, colsample_bytree =0.6, min_child_weight = 1 and subsample = 0.75. |
| Naive Bayes (NB) | laplace = 0, usekernel = TRUE and adjust = 1 |
| k-Nearest Neighbor (KNN) | k = 9 |
| Neural Network (NNET) | size = 1 and decay = 0.0001 |
| Decision Tree (DT) | cp = 0.02632 |
| Logistic Regression (LR) | - |
