# Supplementary material for: An interpretable machine learning approach for predicting drug-resistant epilepsy in children with tuberous sclerosis complex
Source: Front Neurol. 2025 Aug 4;16:1623212. doi: 10.3389/fneur.2025.1623212 (PMC12358403; doi:10.3389/fneur.2025.1623212)
Supplement: Supplementary file 5 [file Table_2.docx]

Supplementary Material

**Supplementary Table 2. Commonly used antiseizure medications (ASMs) in drug-resistant epilepsy (DRE) patients and seizure-free patients**

| **ASM** | **All (%)** | **DRE (n)** | **Seizure-free (n)** |
| --- | --- | --- | --- |
| Vigabatrin | 55 (62.5) | 37 | 18 |
| Valproate | 37 (42.0) | 20 | 17 |
| Oxcarbazepine | 32 (36.4) | 20 | 12 |
| Levetiracetam | 26 (29.5) | 16 | 10 |
| Lamotrigine | 17 (19.3) | 12 | 5 |
| Topiramate | 16 (18.2) | 13 | 3 |
| Lacosamide | 8 (9.1) | 6 | 2 |
| ACTH | 8 (9.1) | 7 | 1 |
| Carbamazepine | 7 (8.0) | 5 | 2 |
| Clonazepam | 5 (5.7) | 5 | 0 |
| Zonisamide | 4 (4.5) | 2 | 2 |
| Clobazam | 3 (3.4) | 3 | 0 |
| Perampanel | 3 (3.4) | 3 | 0 |
| Nitrazepam | 1 (1.1) | 1 | 0 |
| Phenobarbital | 1 (1.1) | 1 | 0 |

ACTH: adrenocorticotropic hormone
