# Supplementary material for: An interpretable machine learning approach for predicting drug-resistant epilepsy in children with tuberous sclerosis complex
Source: Front Neurol. 2025 Aug 4;16:1623212. doi: 10.3389/fneur.2025.1623212 (PMC12358403; doi:10.3389/fneur.2025.1623212)
Supplement: Supplementary file 6 [file Table_3.docx]

Supplementary Material

**Supplementary Table 3. Univariate analysis of drug-resistant epilepsy (DRE) in children with tuberous sclerosis complex (TSC).**

| **variable** | **β** | **SE** | **Z** | **OR(95%CI)** | **p** |
| --- | --- | --- | --- | --- | --- |
| sex |  |  |  |  |  |
| female | 0.000 |  |  | reference |  |
| male | 0.148 | 0.444 | 0.333 | 1.159 (0.483, 2.774) | 0.739 |
| Age of unset | -0.027 | 0.013 | -2.161 | 0.973 (0.946, 0.994) | 0.031 |
| Genotype |  |  |  |  |  |
| *TSC1* | -0.028 | 0.692 | -0.041 | 0.972 (0.246, 3.814) | 0.968 |
| *TSC2* | 0.241 | 0.587 | 0.411 | 1.273 (0.394, 4.055) | 0.681 |
| NMI/ND | 0.000 |  |  | reference |  |
| Family history |  |  |  |  |  |
| no | 0.000 |  |  | reference |  |
| yes | -0.318 | 0.622 | -0.512 | 0.727 (0.209, 2.523) | 0.609 |
| IESS |  |  |  |  |  |
| no | 0.000 |  |  | reference |  |
| yes | 1.729 | 0.506 | 3.416 | 5.636(2.178,16.168) | 0.001 |
| Seizure type at onset |  |  |  |  |  |
| Focal | 0.000 |  |  | reference |  |
| ES | 0.780 | 0.488 | 1.600 | 2.182 (0.849, 5.808) | 0.110 |
| Focal+ES | 2.536 | 1.093 | 2.321 | 12.632(2.139,242.341) | 0.020 |
| Generalized | 0.234 | 1.046 | 0.223 | 1.263 (0.141, 11.335) | 0.823 |
| EEG findings |  |  |  |  |  |
| Normal | 0.000 |  |  | reference |  |
| Focal | 1.761 | 1.110 | 1.586 | 5.818 (0.930, 113.559) | 0.113 |
| Multifocal | 4.522 | 1.292 | 3.501 | 92.000(10.442,2303.642) | <0.001 |
| Generalized | 2.590 | 1.180 | 2.196 | 13.333 (1.805, 281.063) | 0.028 |
| Multiple cortical tubers |  |  |  |  |  |
| no | 0.000 |  |  | reference |  |
| yes | 1.453 | 0.550 | 2.643 | 4.278 (1.511, 13.440) | 0.008 |
| SENs |  |  |  |  |  |
| no | 0.000 |  |  | reference |  |
| yes | 0.941 | 0.465 | 2.025 | 2.563 (1.041, 6.507) | 0.043 |
| SEGAs |  |  |  |  |  |
| no | 0.000 |  |  | reference |  |
| yes | 0.433 | 1.244 | 0.348 | 1.542 (0.142, 33.922) | 0.728 |
| Number of ASMs |  |  |  |  |  |
| <3 | 0.000 |  |  | reference |  |
| ≥3 | 1.805 | 0.479 | 3.766 | 6.078 (2.438, 16.113) | <0.001 |
| mTOR inhibitors |  |  |  |  |  |
| no | 0.000 |  |  | reference |  |
| yes | -0.929 | 0.476 | -1.951 | 0.395 (0.150, 0.982) | 0.051 |
| Developmental delay |  |  |  |  |  |
| no | 0.000 |  |  | reference |  |
| yes | 0.681 | 0.439 | 1.552 | 1.975 (0.841, 4.725) | 0.121 |

DRE: drug-resistant epilepsy; NMI: no mutation identified; ND: not done; IESS: infantile epileptic spasms syndrome; ES: epileptic spasms; EEG: electroencephalogram; SEN: subependymal nodule; SEGA: subependymal giant cell astrocytoma; ASM: anti-seizure medication; mTOR: mechanistic target of rapamycin.
