# Supplementary material for: An interpretable machine learning approach for predicting drug-resistant epilepsy in children with tuberous sclerosis complex
Source: Front Neurol. 2025 Aug 4;16:1623212. doi: 10.3389/fneur.2025.1623212 (PMC12358403; doi:10.3389/fneur.2025.1623212)
Supplement: Supplementary file 7 [file Table_4.docx]

Supplementary Material

**Supplementary Table 4. Performance parameters of the nine machine learning prediction models**

| **Models** | **Sensitivity** | **Specificity** | **Accuracy** | **PPV** | **NPV** | **Kappa score** | **Youden index** |
| --- | --- | --- | --- | --- | --- | --- | --- |
| RF | 0.647 | 0.930 | 0.769 | 0.924 | 0.667 | 0.550 | 0.577 |
| SVM | 0.760 | 0.798 | 0.777 | 0.832 | 0.717 | 0.551 | 0.558 |
| GBM | 0.784 | 0.751 | 0.770 | 0.806 | 0.726 | 0.534 | 0.535 |
| XGB | 0.753 | 0.750 | 0.752 | 0.798 | 0.698 | 0.498 | 0.503 |
| NB | 0.770 | 0.776 | 0.773 | 0.819 | 0.720 | 0.541 | 0.546 |
| KNN | 0.853 | 0.719 | 0.795 | 0.800 | 0.788 | 0.579 | 0.572 |
| NNET | 0.771 | 0.792 | 0.780 | 0.830 | 0.725 | 0.557 | 0.563 |
| DT | 0.600 | 0.632 | 0.614 | 0.682 | 0.545 | 0.227 | 0.232 |
| LR | 0.740 | 0.842 | 0.784 | 0.860 | 0.711 | 0.570 | 0.582 |

RF: random forest, SVM: support vector machine, KNN: K-nearest neighbors, NB: naive bayes, XGB: extreme gradient boosting, GBM: gradient boosting machine, NNET: neural network , DT: decision tree, LR: logistic regression, PPV: **positive predictive value**, NPV: negative predictive value
